# The Potential Association of Later Initiation of Oral/Enteral Nutrition on Euthyroid Sick Syndrome in Burn Patients

**DOI:** 10.1155/2013/707360

**Published:** 2013-01-21

**Authors:** Joaquín Pérez-Guisado, Jesús M. de Haro-Padilla, Luis F. Rioja, Leo C. DeRosier, Jorge I. de la Torre

**Affiliations:** ^1^Service of Plastic, Aesthetic and Reconstructive Surgery, Reina Sofía University Hospital, Avenida Menéndez Pidal s/n, 14004 Córdoba, Spain; ^2^Division of Plastic Surgery, University of Alabama at Birmingham, 510 20th Street S, Birmingham, AL 35294-3411, USA

## Abstract

*Objective*. The aim of this study was to determine if early initiation of oral/enteral nutrition in burn patients minimizes the drop in fT3 levels, reduces the potential for euthyroid sick syndrome (ESS), and shortens the length of hospital stay (LHS). *Subjects and Methods*. We retrospectively evaluated the statistical association of serum fT3, fT4, and TSH at the first (2nd–5th day) and second sample collection (9th–12th day) after the burn injury in 152 burn patients. Three groups were established depending on time of initiation of the oral/enteral nutrition: <24 h before the injury (Group 1), 24–48 h after the injury (Group 2), and >48 h after the injury (Group 3). *Results*. They were expressed as mean ± standard deviation. We found that LHS and the fT3 levels were statistically different in the 3 groups. The LHS (in days) was, respectively, in each group, 16.77 ± 4.56, 21.98 ± 4.86, and 26.06 ± 5.47. Despite the quantifiable drop in fT3, ESS was present only at the first sample collection (2.61 ± 0.92 days) in Group 3, but there was no group with ESS at the second sample collection (9.89 ± 1.01 days). Our data suggest that early initiation of nutritional supplementation decreases the length of hospitalization and is associated with decreasing fT3 serum concentration depression. *Conclusion*. Early initiation of oral/enteral nutrition counteracts ESS and improves the LHS in burn patients.

## 1. Introduction

The oral/enteral route of supplementation is preferable to the parenteral route in most burn patients as it maintains the trophism of the gastrointestinal tract. It promotes the release of intestinal hormones and growth factors, directly nourishes the gastrointestinal tract, and may help reverse the defective gut barrier accompanying burn shock. For these reasons, the oral/enteral route is associated with a decrease in morbidity and mortality in the adult burn population [1].

Additionally, enteral nutrition allows the administration of more balanced and individualized nutritional support. Not only is it associated with lowering infection complications by eliminating catheter contamination, but it is also less costly than parenteral nutrition [2, 3].

In the prognosis of burn patients, the time when nutritional enteral support is started is also paramount. In animal studies, early initiation of enteral feeding decreased high catabolic responses, as well as preventing malnutrition and hypotrophic changes of the gut [4]. The data suggests that in severe burns, enteral feeding started within 6 hours of injury is safe, effective, and reverses several of the most dangerous metabolic and hormonal shifts more quickly [5].

In a patient with a poor nutritional status, nonthyroidal illness syndrome (NTIS), or euthyroid sick syndrome (ESS), has to be considered. The most common feature of ESS is low-T3 syndrome. 

Low-T3 syndrome is frequently observed during caloric restriction, after surgery and after myocardial infarction [6, 7]. This syndrome refers to low peripheral T3 levels in the presence of normal T4 (thyroxine) and TSH (thyroid-stimulating hormone) values. 80% of the circulating T3 is derived from conversion of T4 to T3, which mainly occurs in the liver. The enzyme 5′-deiodinase catalyses the removal of iodine from the outer ring of T4. In sick patients, a decreased 5′-deiodinase activity can be observed. However, an increase in reverse-T3 (rT3), generated by the inner-ring deiodination of thyroxine, is observed. rT3 has no thyromimetic activity. In severely ill patients T4 concentrations also begin to fall in addition to low-T3 concentrations. This hormone constellation has been called low-T3/ low-T4 syndrome and is a bad prognostic indicator within a diagnosis of ESS [8, 9].

Recent studies have shown that ESS is a prognostic indicator of worse outcomes, not only in critically ill patients, but also in burn patients [10]. Furthermore, it has been shown that early enteral nutrition decreases the duration of hospitalization and mortality in the pediatric burn population [11]. However, the link between initiation of oral/enteral nutritional support, the drop in triiodothyronine levels, and its potential influence in ESS have not been studied in an adult burn population. 

Although the American Burn Association practice guidelines state that enteral feedings should be initiated as soon as practical, there is no consensus among physicians concerning the best time to initiate oral/enteral nutrition. Currently, the decision is based on haphazard and subjective criteria developed around the clinical status of the patient. We think this lack of cohesive criteria could result in a longer hospitalization, and a more severe case of ESS or T3 drop. For this reason, we decided to investigate the influence of delayed initiation of oral/enteral nutrition in thyroid hormone levels and the length of hospitalization. 

## 2. Material and Methods

### 2.1. Subject Selection

The information was compiled from clinical histories gathered from the databases of burn patients in Birmingham (Alabama, USA) and Córdoba (Córdoba, Spain) from January 2000 to December 2009. We were looking for patients who fit the inclusion criteria and we grouped them in 3 groups according to the first initiation of oral/enteral nutrition after the burn injury: Group 1 (<24 h), Group 2 (24–48 h), and Group 3 (>48 h).

The chart review was performed by a trained plastic surgeon with long standing experience in chart reviews. 

152 burn patients (93 from Birmingham and 59 from Cordoba) from January 2000 to December 2009 met the inclusion criteria and were selected for our study. All of them survived their injuries. The inclusion criteria were as follows: no preexisting thyroid disease or use of thyroid medications, patients with partial and full thickness burns ranging from 5–20% TBSA (total body surface area), patients under intensive glucose control, age >18 and ≤50 years, no electrical, chemical, or burn associated with major trauma or serious illness, no presence of inhalation injury, Curreri formula applied for the nutrition of the patient, patients that ingested this formula calories prescribed per day, no administration of parenteral nutrition, no administration of dopamine or corticosteroids, samples analyzed over the time with the same thyroid method described ahead for each hospital, and at least two complete thyroid analytical determination at both 2–5 days and 9–12 days after the burn injury. If a patient received more than one thyroid analytical determination during that period, we calculated the mean of those measurements for the collection and analysis of the data.

We considered a minimum of 5% TBSA and a maximum of 20% TBSA, as this was the size threshold capable of being treated at the burn unit of the University Hospital of Reina Sofia. 

We did not include people under 18 years of age since the ethics committee from UAB did not permit it. 

It was not necessary to obtain the authorization of an ethics committee from the University Hospital of Reina Sofia as this was a retrospective study that fulfilled all the terms this institution considered necessary to be excluded from such obligation. Those terms are as follows: the privacy and confidentiality of the patients will be guaranteed, no contact will be made with patients during or after completion of study, all data will be stored on password-protected institutional computers, and no money compensation granted to the research or ownership interest. For the American arm of the study we applied for and received authorization from the institutional review board (IRB) of the University of Alabama at Birmingham (protocol number: X100615005). 

Patient anonymity was preserved in both arms of the study.

### 2.2. Initial Management

On arrival at the burn center, a thorough evaluation was performed and a treatment plan was developed. A thorough history of the burn was obtained, which included the location of the injury, type of agent involved in the burn, duration of exposure to the agent, as well as details of the patient's other comorbidities. The TBSA was calculated and fluids, analgesia, tetanus vaccine, gastrointestinal protection, and subcutaneous heparin were administered.

When calculating TBSA, we only included those areas of partial and full-thickness dermal injury (2° and 3° degree). Superficial burns involving only the epidermis were not included in the calculation. The rule of nines was the most frequent method used for calculating the extent of the burn as it is the most familiar. The fluid resuscitation formula during the first 24 hours following burn injury was the Parkland Formula [12]: 4 cc/kg/%TBSA = total fluid to be administered in the first 24 hours. 50% of fluid should be given in the first 8 hours and the remainder of fluid should be given in the next 16 hours. The fluid is lactated Ringer solution.

At the conclusion of the first 24 hours, we continued fluids to achieve a goal urine output of at least 30 cc/hr until the patient initiated oral tolerance and we considered it clinically indicated to discontinue fluids. 

The administration of fixed amounts of energy to critically ill burn patients based on standardized equations or on the preestablished range of 167–188 kJ/kg day [13] is acceptable only when indirect calorimetry cannot be obtained. We had no means of obtaining indirect/direct calorimetry, and for that reason we applied the well-known Curreri formula. This formula generally overestimates caloric requirements. The Curreri formula is as follows: 25 kcal × weight  (kg) + 40 kcal × %TBSA [12].

Patients received a high protein diet with at least 2 grams of protein per kilogram per day and were supplemented with polyvitamins, minerals, vitamins A, C, and zinc.

The caloric and protein intake was increased by the daily intake of high calorie protein shakes. The minimum number of shakes ingested by each patient was calculated by the hospital nutritionist. From that minimum shakes the patient could consume as many shakes as he wanted to ingest.

### 2.3. Blood Sampling

At least 2 blood samples (weekly measurement) were collected from each patient after admission to the burn center according to the inclusion criteria: at 2–5 days and 9–12 days after the burn injury. 

For the American arm of the study serum fT3, fT4, and TSH levels were measured by chemiluminescent microparticle immunoassay (Architect System, Abbott Laboratories, Diagnostics Division, Abbott Park, IL 60064, USA). Normal ranges in the laboratory were fT3 2.15–4.91 pg/mL; fT4 0.85–1.52 ng/dL; TSH 0.31–4.92 mIU/L.

For the Spanish arm of the study, chemiluminescence (ADVIA Centaur, Bayer Diagnostics) was used to measure serum fT3 (fT3, normal 1.71–3.71 pg/mL), fT4 (fT4, normal 0.70–1.48 ng/dL), and TSH (normal 0.35–4.94 mIU/L).

Both hospital laboratories guaranteed that the hormonal determinations were not affected by serum albumin levels, nonesterified fatty acids, or thyroid binding globulin for these laboratory tests described but not for the tests previously used. These lab tests were validated and implemented in the American arm of the study in 2006 and in the Spanish arm in 2008. For this reason, the samples prior to these dates were not included in this study.

### 2.4. Statistical Analysis

We studied nonthyroid parameters (Table 1) and thyroid parameters (Table 2). Statistical differences between the parameters of each group at first (2nd–5th day) and second sample collection (9th–12th day) were analyzed by one way ANOVA test with SPSS 12.0 (SPSS Inc., Chicago, IL, USA) and are expressed as mean ± standard deviation. If significant differences were observed, Duncan's test was used to determine the number of statistically significant groups. The confidence interval chosen for statistical differences was 95%. The parameters studied were fT3, fT4, and TSH. Before the one way ANOVA test, Kolmogorov-Smirnov and Shapiro-Wilk tests were used for testing normality. The assumption of homoscedasticity was determined with the *F*-Snedecor test.

The non-thyroid parameters considered were (Table 1): the mean of the first and the second sample collections (days), the mean of the first and the second thyroid profile, the mean age, the mean TBSA, the mean length of hospital stay (LHS), and the sex distribution. With the exception of the LHS, the rest of the parameters were studied in order to determine if the distribution was not statistically significantly different in all the groups since it could affect the results. Comparisons between categorical variables were performed with Chi-square test and between noncategorical variables with one way Anova test.

## 3. Results

The study included 152 patients. Table 1 shows the non-thyroid parameters comparing the 3 groups. In this table, the 3 groups were not statistically significantly different at the time of the first and the second sample collection, for sex distribution, age, and TBSA. The length of hospital stay was statistically different in the 3 groups (Table 1), as an earlier initiation of oral/enteral nutrition resulted in a faster discharge from the hospital. There we no deaths in any group.

Having in mind that different fT3 and fT4 assays may report significantly different results, the major concern for this study was the justification for combining results from different assays for fT3 and fT4 depending on the time and the hospital laboratory where the samples were analyzed. For this reason, the results in thyroid parameters for both hospitals during the 3 measurements were compared separately, inside each hospital. As there were no significant differences in both arms of the study for all the parameters examined (*P* > 0.05), included the thyroid parameters, the data of both arms were presented separately and were also pooled and presented together, in order to simplify the analysis.

The results in thyroid parameters (Table 2) during the 3 measurements are presented as follows. The reference intervals described in Table 2 for fT3, fT4, and TSH correspond to the normal ranges given by the laboratories (USA and Spain) and the mean of both laboratories (the third measure).

### 3.1. Period One: 2–5 Days after the Burn Injury (Mean: 2.61 ± 0.92 Days)

As we show in Table 2, fT4 and FSH were not statistically significantly different in the 3 groups. There were significant differences in fT3 levels depending on the group, with higher levels of fT3 in Group 1, and subsequently decreasing in Groups 2 and 3. fT3 levels were normal in Groups 1-2 and below normal in Group 3, evidence that only Group 3 had developed ESS (Table 2).

### 3.2. Period Two: 9–12 Days after the Burn Injury (Mean: 9.89 ± 1.01 Days)

We can see in Table 2 that fT4 and FSH were not statistically significantly different in the 3 groups. Both 3 groups had no ESS due to their normal fT3 levels. Nevertheless both 3 groups were statistically different for fT3 levels, with greater to lesser fT3 levels found in the same order than 2–5 days after the burn injury (Table 2).

## 4. Discussion

Low serum total T3 is the most common abnormality in ESS. It can be observed in about 70% of hospitalized patients [14].

Mechanisms in the pathogenesis of ESS are not yet well understood. Increased levels of endogenous or exogenous glucocorticoids [15], cytokines [16–18], and catecholamines [19] are implicated in the dysregulation of thyroid hormones. These mechanisms occur in conjunction with critical illness and severely hypocaloric diets [20] and favour the conversion of fT4 in rT3 [21]. In our study, those patients with delayed nutritional supplementation showed a higher drop in fT3 levels at 2.61 ± 0.92 days (first period) and at 9.89 ± 1.01 days (second period). The only significant result at the first period was the lowest fT3 levels in patients receiving the most delayed enteral nutrition (>48 h); we should interpret these results with caution, since other variables not considered in the analysis of the data could have a direct impact on the results and act as additional confounding factors. Nevertheless, we have to remind that the 3 groups were not statistically significantly different at the time of the first and the second sample collection, for the most influential variables that we considered in our study: sex distribution, age, and TBSA. 

Although at the second period collection, the fT3 levels were higher in all the groups comparing with the first period. The lowest levels in fT3 for both periods were in Group 3 and the highest in Group 1. Despite this quantifiable drop in fT3, ESS was present just at the first period determination in Group 3, but there was no group with ESS one week later. Maybe the delayed initiation of oral/enteral nutrition in our patients was not long enough to generate a long-term ESS. Based on our findings, we suggest that early initiation of oral/enteral nutrition may diminish the effects of the pathological mechanisms, previously explained, in the pathogenesis of ESS in burn patients. 

Moreover, there were not any deaths in any group. We think this could be due to the inclusion criteria applied; specifically the fact that the severity of injury in the patients assessed was not very high (TBSA <20%). This is supported by a study conducted by Gangemi et al. [10]. They found that a TBSA value >20% is the threshold that largely discriminates (78% of accuracy) between survivors and nonsurvivors.

Recent studies in the paediatric burn population have shown a relationship between later initiation of enteral nutrition and a worsened prognosis [11]. Mosier et al. after a multicenter study found that patients fed early had shorter length of stay and wound infection risk [22]. It has also been shown that a lower fT3 level worsens the prognosis in adult burn patients [10]. In our study, a strong association between delayed initiation of oral/enteral nutrition and increased length of hospitalization was found. TSH and fT4 concentrations, as in our study, are usually normal in patients with ESS [10]. These levels are usually only affected in severely ill patients or a protracted case of ESS, and are correlated with a bad prognosis [8, 9].

The American Burn Association practice guidelines state that enteral feedings should be initiated as soon as practical. Thus, Mosier et al. after their findings, advocate that initiation of enteral nutrition by 24 hours should be used as a formal recommendation in nutrition guidelines for severe burns, and that nutrition guidelines should be actively disseminated to individual burn centers to permit a change in practice [22].

Our results show that burn patients should have to be fed as soon as possible, preferably in the first 24 hours, since it is a cheap measure that would reduce the catabolic changes induced by the burn state and the fasting added, in addition to improve the patient's condition and prognosis, represented by a shorter length of hospital stay. Nevertheless, we have to consider as a limitation of the study that only patients with a modest severity of burns were included in this study. As fasting, itself, can result in the nonthyroidal illness syndrome, even in normal individuals and is rapidly reversed by eating, the decline in thyroid hormone levels in the population of burn patients selected for this analysis may have been primarily due to their nutritional status rather than to the burn injury, itself. Or maybe there was an additive catabolic effect of the fasting and the burn injury, in spite of the fact that it was not severe. Accordingly, the mechanism for nonthyroidal illness in the population of patients selected in this study may not actually be pathological as we suggested, but a normal, physiological, compensatory mechanism. With more severe burn injury, the mechanism for the decline in thyroid function may be much more complicated and under these circumstances, may be seen as pathological. It would be of interest to know whether early nutritional support rapidly recovers thyroid hormone levels in patients with more severe burn injury.

Another potential limitation of the study is that the second measurement was obtained relatively late (mean 9.89 ± 1.01 days), which may have obscured differences between the groups, and the potentiality of selection bias which may have resulted in earlier initiation of enteral feeding in one of the groups.

In conclusion, our results point to a decrease in duration of hospitalization with the early initiation of oral/enteral nutrition, as well as a decrease in fT3 serum concentration depression. By doing so, early oral/enteral nutrition counteracts ESS and improves the short-term prognosis of burn patients.

## Figures and Tables

**Table 1:** Nonthyroid parameters.

Parameters	Group 1	Group 2	Group 3	Overall mean	*Significance
1st sample collection (days)	2.55 ± 0.86	2.67 ± 0.97	2.63 ± 1.00	2.61 ± 0.92	*P > 0.05
2nd sample collection (days)	9.87 ± 1.01	9.96 ± 1.07	9.83 ± 0.95	9.89 ± 1.01	*P > 0.05
Age (18–50 years old)	35.20 ± 7.56	37.17 ± 9.70	35.74 ± 8.64	35.92 ± 8.49	*P > 0.05
TBSA	12.93 ± 3.99	13.24 ± 4.79	13.46 ± 2.92	13.14 ± 4.02	*P > 0.05
LHS	16.77 ± 4.56	21.98 ± 4.86	26.06 ± 5.47	20.49 ± 6.14	*P < 0.05; A, B, C*
Sex: male/female	0.75/0.25	0.76/0.24	0.80/0.20	0.76/0.24	*P > 0.05
*N* (males/females)	71 (53/18)	46 (35/11)	35 (28/7)		

*The A, B, and C Duncan's groups indicate that they are statistically different. Data are expressed as mean ± standard deviation.

**Table 2:** Thyroid parameters.

Groups: parameters	2–5 days after	Duncan	9–12 days after	Duncan
	USA 2.15–4.91 pg/mL		USA 2.15–4.91 pg/mL	
fT3 reference interval	SPAIN 1.71–3.71 pg/mL		SPAIN 1.71–3.71 pg/mL	
	Mean 1.98–4.44 pg/mL		Mean 1.98–4.44 pg/mL	

	USA 2.42 ± 0.16 pg/mL	A	USA 2.76 ± 0.20 pg/mL	A
Group 1: fT3	SPAIN 1.86 ± 0.22 pg/mL	A	SPAIN 2.40 ± 0.23 pg/mL	A
	Mean 2.20 ± 0.20 pg/mL	A	Mean 2.62 ± 0.21 pg/mL	A

	USA 2.31 ± 0.17 pg/mL	B	USA 2.57 ± 0.15 pg/mL	B
Group 2: fT3	SPAIN 1.82 ± 0.21 pg/mL	B	SPAIN 1.95 ± 0.20 pg/mL	B
	Mean 2.12 ± 0.19 pg/mL	B	Mean 2.33 ± 0.17 pg/mL	B

	USA *1.63 ± 0.19 pg/mL	C	USA 2.27 ± 0.16 pg/mL	C
Group 3: fT3	SPAIN *1.26 ± 0.23 pg/mL	C	SPAIN 1.83 ± 0.20 pg/mL	C
	Mean *1.49 ± 0.21 pg/mL	C	Mean 2.10 ± 0.18 pg/mL	C

	USA 0.85–1.52 ng/dL		USA 0.85–1.52 ng/dL	
fT4 reference interval	SPAIN 0.70–1.48 ng/dL		SPAIN 0.70–1.48 ng/dL	
	Mean 0.79–1.50 ng/dL		Mean 0.79–1.50 ng/dL	

	USA 1.20 ± 0.20 ng/dL	A	USA 1.27 ± 0.14 ng/dL	A
Group 1: fT4	SPAIN 0.85 ± 0.30 ng/dL	A	SPAIN 1.10 ± 0.18 ng/dL	A
	Mean 1.07 ± 0.25 ng/dL	A	Mean 1.21 ± 0.16 ng/dL	A

	USA 1.18 ± 0.17 ng/dL	A	USA 1.26 ± 0.8 ng/dL	A
Group 2: fT4	SPAIN 0.83 ± 0.22 ng/dL	A	SPAIN 1.07 ± 0.13 ng/dL	A
	Mean 1.04 ± 0.19 ng/dL	A	Mean 1.19 ± 0.10 ng/dL	A

	USA 1.14 ± 0.10 ng/dL	A	USA 1.25 ± 0.10 ng/dL	A
Group 3: fT4	SPAIN 0.80 ± 0.16 ng/dL	A	SPAIN 1.05 ± 0.14 ng/dL	A
	Mean 1.01 ± 0.13 ng/dL	A	Mean 1.17 ± 0.12 ng/dL	A

	USA 0.31–4.92 mIU/L		USA 0.31–4.92 mIU/L	
TSH reference interval	SPAIN 0.35–4.94 mIU/L		SPAIN 0.35–4.94 mIU/L	
	Mean 0.33–4.93 mIU/L		Mean 0.33–4.93 mIU/L	

	USA 4.13 ± 0.18 mIU/L	A	USA 3.05 ± 0.15 mIU/L	A
Group 1: TSH	SPAIN 4.15 ± 0.24 mIU/L	A	SPAIN 3.10 ± 0.21 mIU/L	A
	Mean 4.14 ± 0.21 mIU/L	A	Mean 3.07 ± 0.18 mIU/L	A

	USA 4.21 ± 0.14 mIU/L	A	USA 3.08 ± 0.18 mIU/L	A
Group 2: TSH	SPAIN 4.10 ± 0.20 mIU/L	A	SPAIN 3.12 ± 0.23 mIU/L	A
	Mean 4.17 ± 0.17 mIU/L	A	Mean 3.08 ± 0.21 mIU/L	A

	USA 4.27 ± 0.6 mIU/L	A	USA 3.14 ± 0.07 mIU/L	A
Group 3: TSH	SPAIN 4.06 ± 0.15 mIU/L	A	SPAIN 3.17 ± 0.14 mIU/L	A
	Mean 4.19 ± 0.10 mIU/L	A	Mean 3.15 ± 0.10 mIU/L	A

*The Duncan test has been applied for the variation of each parameter (fT3, fT4, and TSH) inside the measurement time. The same letter inside the same measurement time indicates no statistical differences. Data are expressed as mean ± standard deviation.
